# Secondary Structure Changes in ApoA-I Milano (R173C) Are Not Accompanied by a Decrease in Protein Stability or Solubility

**DOI:** 10.1371/journal.pone.0096150

**Published:** 2014-04-22

**Authors:** Jitka Petrlova, Jonathan Dalla-Riva, Matthias Mörgelin, Maria Lindahl, Ewa Krupinska, Karin G. Stenkula, John C. Voss, Jens O. Lagerstedt

**Affiliations:** 1 Department of Experimental Medical Science, Lund University, Lund, Sweden; 2 Department of Infection Medicine, Lund University, Lund, Sweden; 3 School of Medicine, University of California Davis, Davis, California, United States of America; University of Pittsburgh School of Medicine, United States of America

## Abstract

Apolipoprotein A-I (apoA-I) is the main protein of high-density lipoprotein (HDL) and a principal mediator of the reverse cholesterol transfer pathway. Variants of apoA-I have been shown to be associated with hereditary amyloidosis. We previously characterized the G26R and L178H variants that both possess decreased stability and increased fibril formation propensity. Here we investigate the Milano variant of apoAI (R173C; apoAI-M), which despite association with low plasma levels of HDL leads to low prevalence of cardiovascular disease in carriers of this mutation. The R173C substitution is located to a region (residues 170 to 178) that contains several fibrillogenic apoA-I variants, including the L178H variant, and therefore we investigated a potential fibrillogenic property of the apoAI-M protein. Despite the fact that apoAI-M shared several features with the L178H variant regarding increased helical content and low degree of ThT binding during prolonged incubation in physiological buffer, our electron microscopy analysis revealed no formation of fibrils. These results suggest that mutations inducing secondary structural changes may be beneficial in cases where fibril formation does not occur.

## Introduction

Apolipoprotein A-I (apoA-I) is the main protein of high-density lipoprotein (HDL) and mediates efflux of cellular cholesterol from the peripheral tissues to the liver for excretion from the body in feces [1]. This transport process, the so-called reverse cholesterol transfer (RCT) pathway, involves a number of participating membrane proteins and plasma enzymes including ATP-binding cassette transporters A1 and G1 (ABCA1 and ABCG1), scavenger receptor BI (SR-BI) [2], [3], and lecithin cholesterol-acyl transferase enzyme (LCAT), the latter being associated with maturation of HDL in plasma [4]. In addition, HDL is involved in anti-inflammatory and anti-oxidant processes that occur through non-RCT pathways [5], [6].

Several variants of apoA-I with altered functionality have been identified. The first naturally occurring variant of apoA-I described was the apoA-I Milano (apoAI-M) variant, which was identified in a family originating from the village of Limone sul Garda in northern Italy [7]. The single mutation of this variant results in a substitution of Arg to Cys in the primary structure at residue 173 [8]. Described carriers of the Milano variant of apoA-I are heterozygotes and have very low plasma levels of apoA-I and HDL cholesterol as well as normal or moderately elevated plasma triglycerides [9]. Despite this pro-arteriosclerotic lipoprotein profile, carriers of the apoAI-M variant display no increase in cardiovascular disease or events at the preclinical level [10]. In fact, the RCT capacity of apoAI-M carriers is enhanced and the variant also exhibits anti-inflammatory and plaque stabilizing properties [11]. The beneficial effect of infusion of recombinant apoAI-M has been shown by reduction of atherosclerotic lesions in experimental animal models [12], [13]. Clinical trials have also demonstrated a reduction of atheromas after repeated administration of apoAI-M/phospholipid complexes to patients with coronary disease [14], [15]. Clearly, the Milano variant provides positive effects on the cardiovascular system.

However, the location of the R173C amino acid substitution is in a region of the apoA-I primary structure that is known to harbor several fibrillogenic variants (i.e., variants that form fibrils composed of beta-sheet rich amyloid structure, or other type of fibril-structure), which lead to tissue specific plaque formation of the fibrillogenic protein and consequent organ failure [16]–[18]. Considering the location of the amino acid substitution to this region and that the Milano variant is currently under investigation as an infusion therapy in cardiovascular disease, we wished to understand its susceptibility to aggregation. We have here examined the intrinsic propensity of the apoAI-M variant to aggregate into fibrils.

## Materials and Methods

### Production of Recombinant Protein

A bacterial expression system consisting of pNFXex plasmid in *Escherichia coli* strain BL21(DE3) pLysS cells (Invitrogen) was used to produce the apoAI-WT and apoAI-M proteins, as previously described [16], [19]. Primer-directed PCR mutagenesis was used to create the R173C mutation. The mutation was verified by dideoxy automated fluorescent sequencing (GATC Biotech). After purification of apoA-I proteins on Ni^2+^-chelated columns (GE Healthcare) and desalting to remove imidazole, Tobacco etch virus (TEV) protease treatment was employed to cleave the His-tag. This was followed by a second Ni^2+^- column passage where the TEV protease and the cleaved His-tag were retained on the column. The flow-through containing cleaved apoA-I proteins was desalted into phosphate buffered saline, pH 7.4, 150 mM NaCl, concentrated with 10 kDa molecular weight cut-off Amicon Ultra centrifugal filter devices (Millipore) and stored at 4°C prior to use. Protein purity was confirmed by sodium dodecyl sulfate (SDS)-polyacrylamide gel electrophoresis with Coomassie blue staining and protein concentrations were determined by use of a Nanodrop 2000c spectrophotometer (Thermo scientific).

### Limited Proteolysis

Protein (5 µg) in phosphate buffered saline, pH 7.4, 150 mM NaCl, was treated with 1∶2000 ratio (wt/wt) of high purity chymotrypsin (Sigma-Aldrich #C3142) for the indicated periods of times. Reactions were stopped with protease inhibitor cocktail (Roche #05892791001) followed by addition of SDS loading buffer. Samples were stored at −20°C until analysis with SDS-PAGE.

### Circular Dichroism Spectroscopy

Circular dichroism spectroscopy (CD) measurements were performed on a Jasco J-810 spectropolarimeter equipped with a Jasco CDF-426S Peltier set to 25°C. Averages of five scans were baseline-subtracted (PBS buffer; 25 mM phosphate, 150 mM NaCl) and the alpha-helical content was calculated from the molar ellipticity at 222 nm as previously described [16].

For thermal stability experiments, spectra were obtained from 25°C to 80°C with 2.5°C increments. ApoA-I was diluted to 0.2 mg/ml in PBS (final concentration was 25 mM phosphate, 150 mM NaCl, pH 7.4), placed in a 1 mm quartz cuvette and, after extensive purging with nitrogen, scanned in the region 200 to 260 nm (scan speed was 20 nm/min). The Boltzmann function within the GraphPad software (GraphPad Software, Inc., CA, USA) was used to fit the molar ellipticity values at 222 nm of the temperature gradient to a sigmoidal fit curve.

### Thioflavin T (ThT) Binding Assay

ApoAI-M, apoAI-WT and apoAI-Iowa(G26R) variant (0.2 mg/ml) were incubated at 37°C and diluted with ThT stock solution at time of use. 180 µl of protein was incubated for 10 min in the dark with 20 µl of a ThT (100 µM)/glycine (10 mM) solution (ThT stock: 1 mM stored in the dark at 4°C; Glycine buffer stock: 0.1 M at pH 8.5 stored at 4°C). ThT fluorescence was then measured using a VICTOR3 Multilabel Plate Counter (PerkinElmer, Waltham, MA, USA) spectrofluorometer at an excitation wavelength of 450 nm and an emission wavelength of 545 nm, with excitation and emission slit widths of 10 nm [16].

### Electron Microscopy

Protein samples incubated at 37°C for 30 days were diluted and analyzed by negative stain electron microscopy as described previously [20]. Five microliter aliquots were adsorbed onto carbon-coated grids for 1 min, washed with two drops of water, and stained on two drops of 0.75% uranyl formate. The grids were rendered hydrophilic by glow discharge at low pressure in air. Specimens were observed in a JEOL JEM 1230 electron microscope operated at 80 kV accelerating voltage, and images were recorded with a Gatan Multiscan 791 CCD camera [16]. Control experiments comparing apoA-I-WT and apoA-I-Iowa are shown in Figure S1.

### ApoA-I in vivo Analysis

Male C57/Bl6 mice purchased from Taconic (Ry, Denmark) were used at the age of 10–11 weeks. Mice fasted overnight (12 h) were injected intraperitoneally (i.p.) with apoAI-WT or apoAI-M (14 mg/kg) (control animals received NaCl). Blood samples were collected three hours following treatment. Serum samples (2 µL) were separated by SDS-PAGE, in the presence or absence of the reducing agent dithiothreitol (Sigma), and transferred to nitrocellulose membranes, probed with anti-human apoA-I antibodies (Abcam) and immune detection performed with HRP-conjugated secondary antibodies (GE Healthcare). Blots were imaged using the Odyssey Fc system (LI-COR) and quantified using Image studio v2.0 software. The animal procedures were approved by the *Malmö/Lund Committee for Animal Experiment Ethics*.

## Results and Discussion

### Quality Assessment of apoAI-M Protein

**Figure 1:**
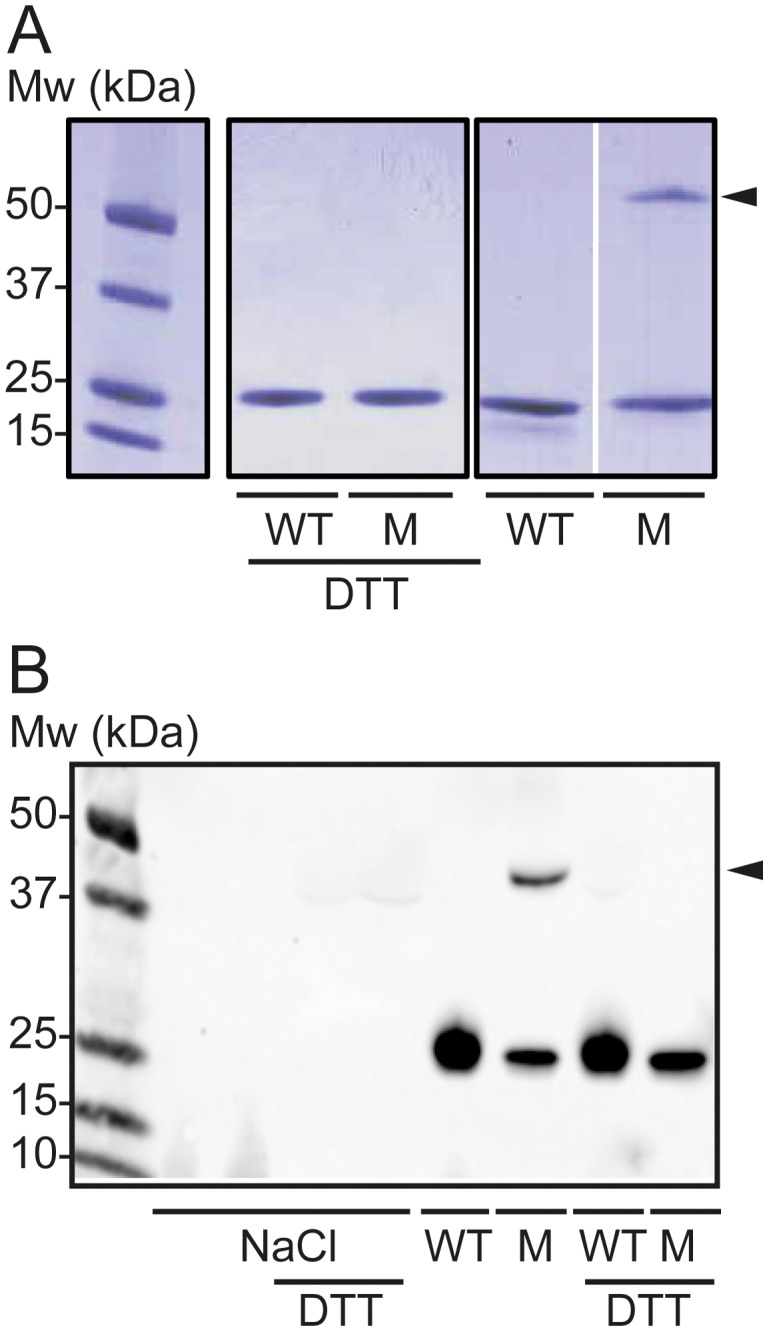
Covalent Cys-Cys binding and dimer formation of ApoAI-M. *A*, Purified apoAI-M (M) and apoAI-WT (WT) proteins (2 µg) were analyzed by SDS-PAGE (4–15% Tris-glycine) in the presence or absence of the reducing agent DTT. Formed apoAI-M dimers are indicated (*arrow*). *B*, Western blot analysis of apoAI-M (M) and apoAI-WT (WT) proteins in plasma samples from mice treated with the respective apoA-I protein. The SDS-PAGE separation was performed in the presence or absence of the reducing agent DTT to distinguish protein in covalently attached Cys-Cys dimers (*arrow*). Analysis of mouse plasma from control animals treated with saline (NaCl) was included to show specificity of the antibodies for human apoA-I protein. Data shown is representative of three experiments/animals.

Although the structural basis for the positive effect of the Milano mutation on cardiovascular health is unclear, the protein is known to form disulfide-linked dimers via R173C [21], [22]. We therefore carried out analyses to ensure adequate protein purity as well as functional Cys-Cys-linked dimer formation of apoAI-M. SDS-PAGE analysis in the absence or presence of reducing agent was used to detect Cys-Cys-linked dimer formation of the human apoAI-M protein. As can be seen in Figure 1, purified apoAI-M protein formed covalently attached dimers (arrow in Figure 1A) that can be separated with the addition of a reducing agent, whereas apoAI-WT proteins did not form covalent bonds. To confirm that the *in vitro* analyses of the proteins represent the *in vivo* oligomeric organization, human apoAI-WT and apoAI-M proteins were injected intraperitoneally in mice followed by serum sampling at 3 hours post-injection. The serum samples were separated on SDS-PAGE in the presence and absence of reducing agent followed by western blot analysis with antibodies specific for human apoA-I protein. The antibodies used do not detect mouse apoA-I (see negative NaCl control in Figure 1B). The results showed that Cys-Cys-linked apoAI-M dimers were also present *in vivo* (arrow in Figure 1B). The results are in agreement with earlier studies that describe the presence of monomer and homo-dimer in human plasma of apoAI-M carriers and in the plasma of a mouse model expressing human apoAI-M [21], [22]. Finally, native gel separation followed by western blot analysis of the serum samples shows that both apoAI-M and apoAI-WT are fully lipidated 3 hours post-injection, and have formed lipid-protein complexes of comparable sizes (not shown). In conclusion, the produced human apoAI-M protein forms disulfide-linkages *in vitro* and *in vivo*, and is capable of assembly into HDL particles.

### Comparison of Susceptibility to Proteolytic Cleavage

**Figure 2:**
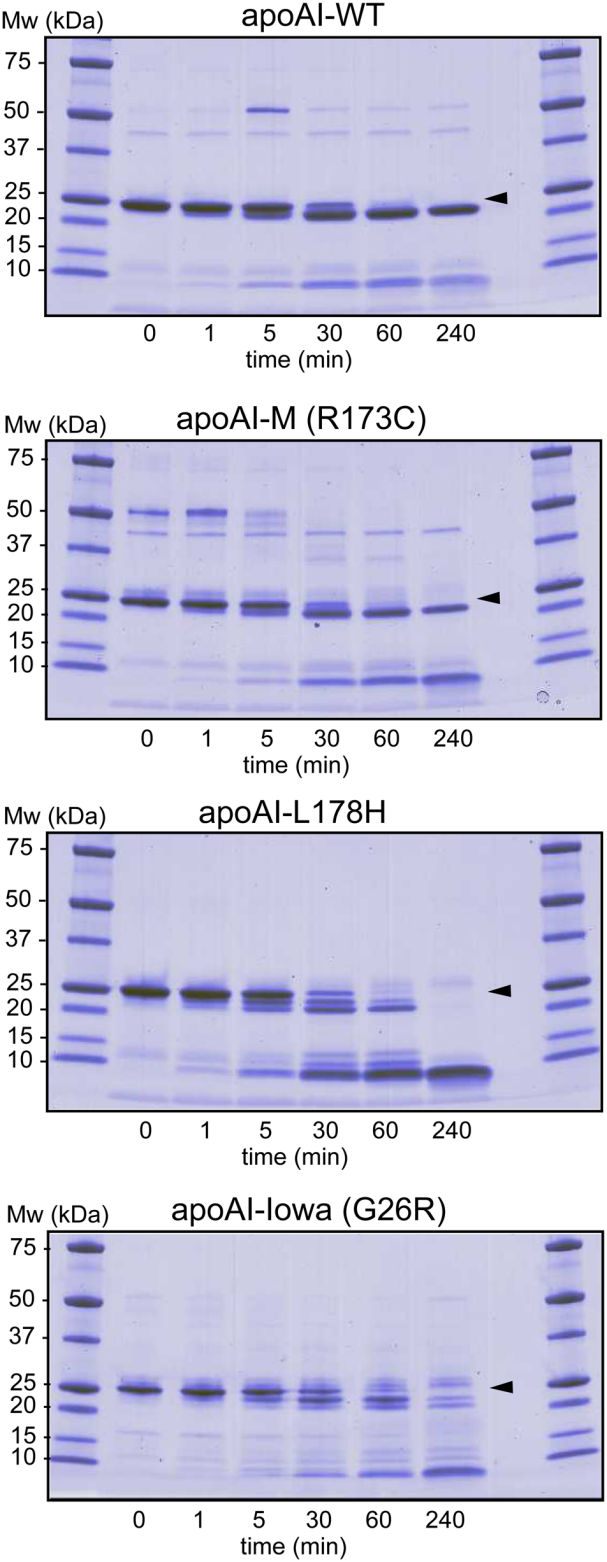
Limited proteolysis of apoA-I proteins to assay for structure accessibility. ApoA-I proteins (0.3 mg/ml) were incubated with chymotrypsin at 37°C for indicated times followed by SDS-PAGE separation and coomassie staining of the gel. *Arrows* indicate migration distance of full-length proteins.

Our earlier analyses showed that the L178H and G26R mutations lead to increased protease sensitivity in the N-terminus (16, 18). We here used limited proteolysis to investigate if also the R173C substitution led to increased susceptibility to proteolytic cleavage. Figure 2 shows protein and protein fragments of apoAI-WT, apoAI-M, apoAI-L178H, and apoAI-Iowa (G26R) after incubation with chymotrypsin at indicated times. In agreement with earlier findings, chymotrypsin cleaves apoAI-WT at one main site leading to a protein fragment corresponding to residues 1–225 that is stable also after 240 min of incubation (18). Similarly, chymotrypsin treatment of apoAI-M resulted in one major cut in the primary structure leading to stable protein fragments of comparable sizes as the WT protein. In contrast, proteolytic cleavage of the G26R and L178H proteins led to an array of peptide fragments, and the case of L178H, eventually to complete degradation of the protein. Based on the findings we conclude that the R173C substitution is more protected than G26R and L178H to limited proteolysis, but not different to apoAI-WT.

### Changes in the Secondary Structure of apoAI-M as Determined by CD

We have previously shown that the L178H variant aggregates and form fibrils via a process that includes substantial increases in alpha helical content of the protein (from about 50% helical structure in the native, non-aggregated protein to about 80% helical structure after fibrillization) [16]. Given the close proximity of R173C to amino acid residue 178 in the primary structure, we tested whether the R173C substitution also results in a time-dependent increase of alpha helical structure.

**Figure 3:**
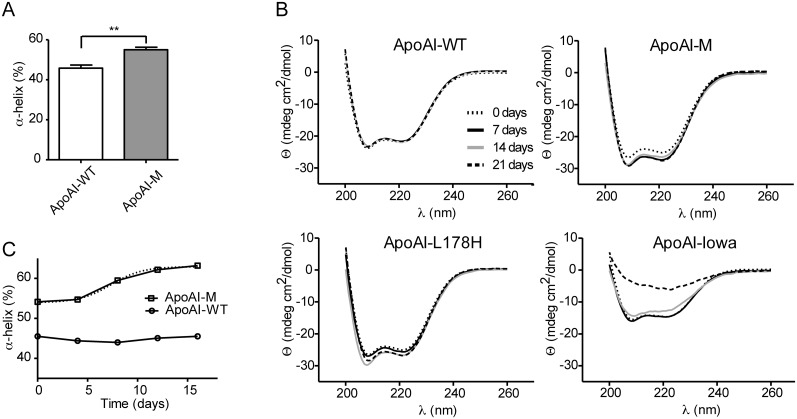
Structural transitions of apoA-I proteins assayed by CD spectroscopy. *A*, The alpha helical content was calculated from the value of molar ellipticity at the wavelength 222 nm at the time point 0 days for apoAI-WT and apoAI-M (25°C). *B*, Circular dichroism spectroscopy was used to analyze secondary structure changes over time. Scans ranging from 200 nm to 260 nm of apoAI-Iowa (G26R), apoAI-Milano (R173C), apoAI-L178H and apoAI-WT proteins (at concentrations of 0.2 mg/ml) incubated at 37°C for up to 21 days (0, 7, 14 and 21 days of incubation) are shown. While the secondary structure of apoAI-WT protein is unchanged during the time course, the spectral changes of the apoAI-Milano and the apoAI-L178H proteins indicate increased alpha-helical content (as indicated by an increase in molar ellipticity at 222 nm), whereas the amyloidogenic apoAI-Iowa displays a reduction in alpha helical secondary structure with time. *C,* The percentage of alpha helix was measured during 16 days of incubation at 37°C at different time points (0, 4, 8, 12 and 16 days). Boltzmann function was used to determinate the transition time of the apoAI-Milano variant. ApoAI-WT did not exhibit any significant changes in the alpha helical content when incubated at identical conditions during same time period. **p<0.01, n = 3.

Circular dichroism (CD) spectroscopy was first used to estimate the secondary structure content of the purified apoAI-M and apoAI-WT proteins in solution at a concentration of 0.2 mg/ml at time zero. Using the 222 nm values the helical content was estimated to 55.0±1.3% and 45.8±1.6% (SEM; n = 3; p<0.01) at 25°C for apoAI-M and apoAI-WT, respectively (Figure 3A). This is in good agreement with Suurkkuss et al. [23] who reported an alpha-helical content of apoAI-M to about 50–59% (depending on oligomeric state), and with Alexander et al. [22] who reported an alpha-helical content of 44±4% for apoAI-WT.

CD analysis was then performed on 0.2 mg/ml of apoAI-M and apoAI-WT during a 3-week incubation time period at 37°C in PBS buffer, pH 7.4 (Figure 3B). The apoA-I variants Iowa (G26R) [18] and L178H [16], which are both linked to hereditary amyloidosis [17], [24] and are known to display increased content of beta-strand structure and alpha helical structure, respectively, under these conditions were used as controls. While the CD spectra for wild-type protein was unchanged throughout the time course (Figure 3B, upper left), the Milano (R173C) variant showed a change in spectra that corresponds to an increase in the alpha helical content (Figure 3B, upper right). The t_1/2_ for this change was about 8 days (alpha helical content is plotted in Figure 3C as a function of time), which is significantly shorter than that previously reported for the L178H variant (≈12 days) [16], and as shown here (Figure 3B, lower left). As expected the Iowa variant displayed CD spectra that correspond to significant beta-strand structure content (Figure 2B, lower right).

The structures of aggregation-prone variants of apoA-I are typically less stable than wild-type apoA-I protein. CD spectroscopy was therefore used to determine the thermal stability of apoAI-M compared to apoAI-WT, which was compared to our published results on the L178H variant [16]. Unfolding of apoAI-M and apoAI-WT caused by step-wise increase of temperature resulted in sigmoidal, monophasic transition with an apparent T_m_ 50.9±1.4°C and T_m_ 55.9±1.4°C (SEM; n = 3), respectively. The difference was not significant (not shown). The apparent thermal stability of the Milano variant was similar to those previously described (T_m_≈53°C in [22], [23]), whereas the apoA-I-WT Tm was slightly lower compared to earlier analyses using CD spectroscopy (T_m_≈58–60°C in [22], [23], [25], [26], [27]) and higher or comparable to those determined by calorimetry (T_m_≈52–57°C in [27], [28]). The finding that the Tm of apoAI-M is clearly higher than that of L178H (45±0.6°C; as previously described in [16]) suggests that the faster conversion to alpha helical structure of the apoAI-M variant is not due to decreased protein stability.

### Low Affinity of the Amyloidophilic Dye Thioflavin T to Milano Variant

**Figure 4:**
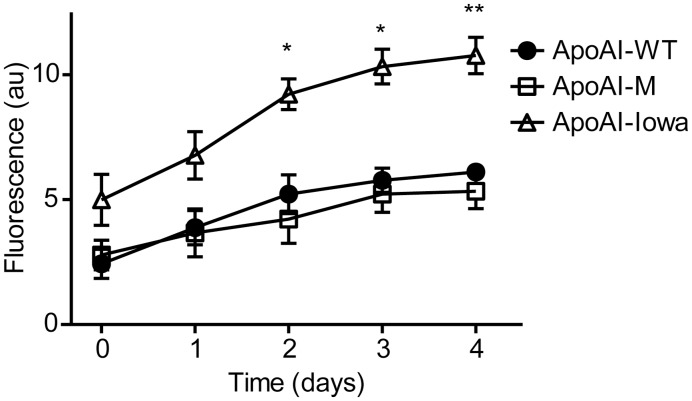
The amyloidophilic dye ThT does not bind to the apoAI-Milano protein. The binding of thioflavin T (ThT) to apoAI-M was assessed and compared to apoAI-WT. No significant binding of ThT to apoAI-WT and apoAI-M was observed over a four-week period of incubation at 37°C. This result is in contrast to apoAI-Iowa protein, known for its propensity to form cross-beta amyloids, which had higher ThT binding than both apoAI-WT and apoAI-M, and thus higher beta sheet content. *p<0.05, n = 3; **p<0.01, n = 3.

We next analyzed if the secondary structure conversion of the Milano variant was associated with formation of beta sheet containing amyloids (Figure 4). Thioflavin T (ThT) is a fluorescent dye used to study the amyloidogenic properties of proteins by specifically binding to beta sheet structure of amyloid fibrils with resulting increase in fluorescence. In this experiment we compared ThT binding to apoAI-M with apoAI-WT and the amyloidogenic Iowa variant (G26R) as negative and positive controls respectively. The results show that while the Iowa variant increasingly binds ThT during the time course, the apoAI-M has approximately the same low binding affinity to ThT as apoAI-WT. We therefore conclude that amino acid substitution from Arg to Cys at residue 173 of the apoAI-M protein does not lead to an elevated intrinsic propensity to form beta-sheet containing amyloid.

### Negative Stain Electron Microscopy Analysis

**Figure 5:**
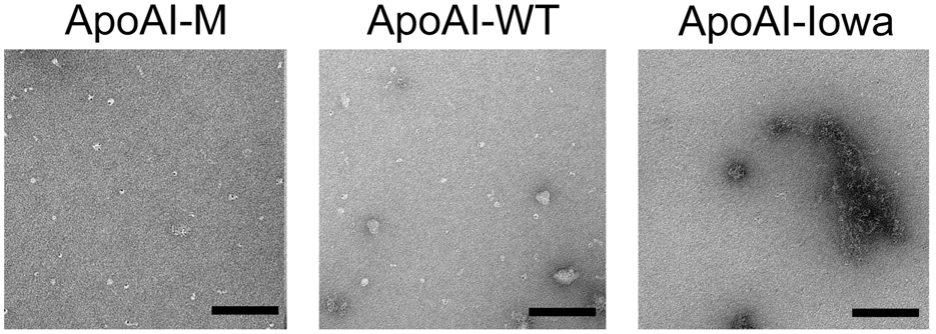
Electron microscopy (negative stain) analysis shows no fibril formation of apoA-I-M after four weeks of incubation at 37°C. ApoAI-Milano (R173C), apoAI-WT and apoAI-Iowa (G26R) proteins were incubated at 37°C for four weeks followed by negative stain EM analysis. While the positive control apoAI-Iowa formed elongated fibrils (right panel), neither apoAI-WT (middle panel) nor apoAI-M (left panel) displayed any fibril formation. Size bars are 100 nm.

Our previous analyses on the L178H variant showed formation of twisted, helical fibrils (with a diameter of about 10 nm and with lengths ranging from 30 to 120 nm) despite no specific increase in binding to ThT [16]. We reasoned that the R173C variant would possibly form similar helical fibrils that were undetected by the beta-amyloid specific ThT dye. Negative stain electron microscopy (EM) was therefore used to analyze for a potential formation of apoAI-M fibrils. Milano variant and wild-type protein were incubated in PBS buffer at 37°C for 4 weeks followed by dilution in tris-buffer saline (pH 7.4) and then analyzed by EM. As can be seen in Figure 5 (left panel), rounded molecular aggregations, but no elongated fibrils, were observed for the apoAI-M samples, which was consistent with the appearance of the incubated apoAI-WT protein (Figure 5, middle panel). Similar structures of apoA-I-WT were previously shown by Ramella et al [29] when incubated at physiological conditions. As a positive control for aggregate formation, the amyloidogenic variant apoAI-Iowa was used, which exhibited a strong propensity to aggregate as shown by formation of elongated pre-fibrillar structures and aggregates (Figure 5, right panel; Figure S1). Thus, in contrast to the L178H variant, the apoAI-M protein does not form fibrils under the experimental conditions used.

## Conclusions

Our data suggest that despite the fact that the apoAI-M protein shares several features with the L178H variant, including increased helical secondary structure formation during incubation, the R173C substitution does not carry an intrinsic propensity to form fibrils and/or amorphous aggregates. The finding is partly unexpected as the mutation is located to a domain of the apoA-I structure where amino acid substitution can result in increased susceptibility to proteolysis and/or subsequent fibril formation. This may be explained by the ability of apoAI-M to maintain protein stability via covalent disulphide bridge interaction. Another distinct difference between the two variants is the change in charge of the side-chains (hydrophobic to basic in L178H and basic to neutral in R173C), which may be a contributing factor to the observed differences. In addition, the increase in helical structure can potentially be attributed to an increase in coiled-coil formation in the dimeric organization of the protein, which is likely induced by the –S-S- covalent bonds between the proteins. Therefore, the occurrence of disulfide bridging by Milano proteins may not only result in a therapeutically-beneficial form of the protein, but may also prevent the formation of large fibril assemblies, which would likely result in a pathogenic state.

Proteases, e.g. chymase and tryptase [30], are expected to be crucial for the maturation of fibrils as N-terminal fragments (the first 80–95 amino acids of the extreme N-terminal domain) are commonly found in plaques [31]. However, there are also examples of variants/conditions that lead to aggregation of full-length apoA-I. Those include the presence of full-length apoA-I protein in plaques of humans carrying the L178H variant [17], aggregation of apoA-I-WT at low pH [29], and fibril-formation of apoA-I-WT following methionine oxidation [25]. Thus, while our study defines a lack of intrinsic propensity of the apoAI-M protein to form fibrils *in vitro*, further experimental work will be needed to analyze the potential role of extrinsic factors *in vivo* (plasma proteins, proteases, extracellular matrix components, *etc*.) on apoAI-M aggregation propensity.

## Supporting Information

Figure S1 Transmission electron micoscopy (TEM) images of WT and IOWA apoA-I. WT (A) and IOWA (B) apoA-I proteins at a protein concentration of 0.2 mg/ml were incubated at 37 C for 28 days followed by TEM analyses. Size bars are 100 nm. (black bars) or 2 µm (white bars).
(PDF)
