# Supplementary material for: Secondary Structure Changes in ApoA-I Milano (R173C) Are Not Accompanied by a Decrease in Protein Stability or Solubility
Source: PLoS One. 2014 Apr 22;9(4):e96150. doi: 10.1371/journal.pone.0096150 (PMC3995965; doi:10.1371/journal.pone.0096150)
Supplement: Figure S1 — Transmission electron micoscopy (TEM) images of WT and IOWA apoA-I. WT (A) and IOWA (B) apoA-I proteins at a protein concentration of 0.2 mg/ml were incubated at 37 C for 28 days followed by TEM analyses. Size bars are 100 nm. (black bars) or 2 µm (white bars). (PDF) [file pone.0096150.s001.pdf]

A

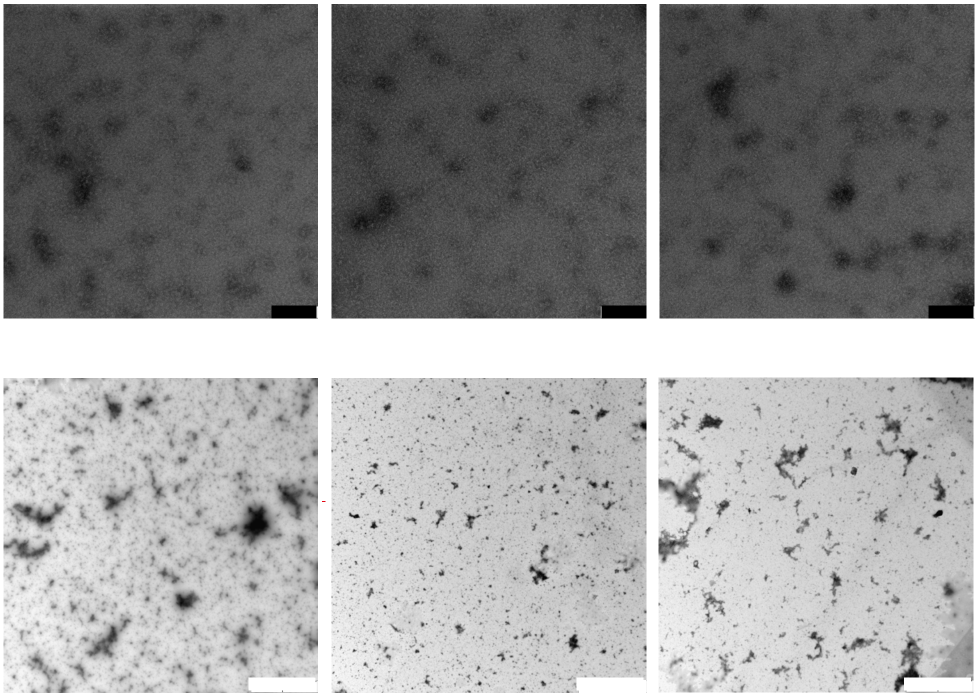

B

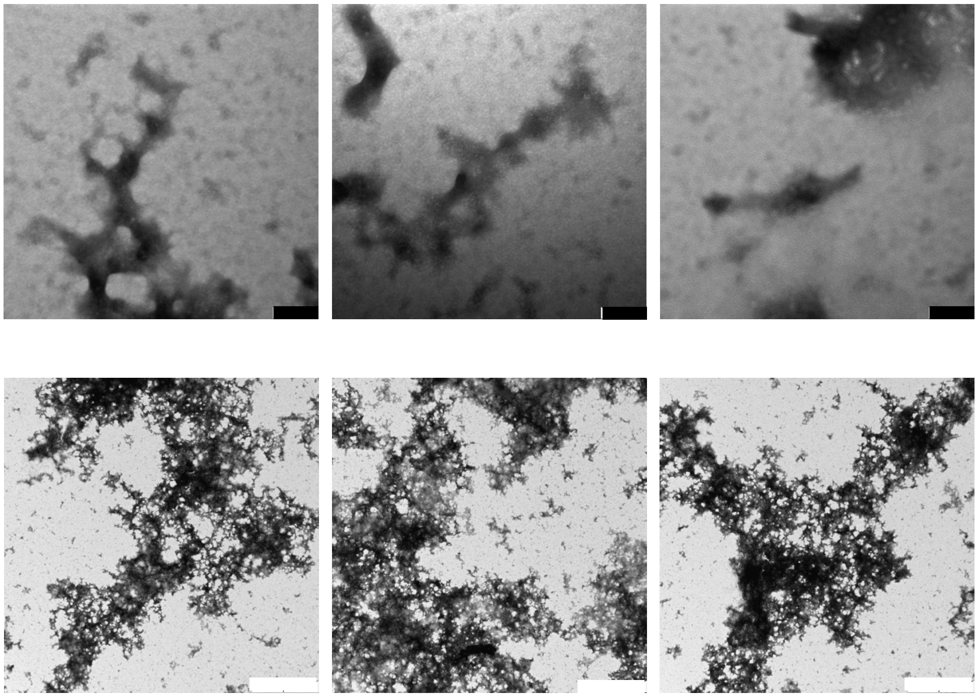

Figure S1. Transmission electron micoscopy (TEM) images of WT and IOWA apoA-I. WT (A) and IOWA (B) apoA-I proteins at a protein concentration of 0.2 mg/ml were incubated at 37 C for 28 days followed by TEM analyses. Size bars are 100 nm (black bars) or 2 μm (white bars).
